# (*Z*)-2-[(4-Methyl­phen­yl)sulfon­yl]-1,2-diphenyl­ethene­selenol

**DOI:** 10.1107/S1600536812001638

**Published:** 2012-01-18

**Authors:** Sampath Natarajan, Rita Mathews

**Affiliations:** a Department of Advanced Technology Fusion, Konkuk University, 1 Hwayang-dong, Gwangjin-gu, Seoul 143 701, Republic of Korea

## Abstract

In the title compound, C_21_H_18_O_2_SSe, the dihedral angle between the *cis* phenyl rings is 64.3 (1)° and those between the toluene and the phenyl rings are 21.1 (2) and 72.0 (1)°, respectively. An intra­molecular Se—H⋯O hydrogen bond occurs. In the crystal, mol­ecules are connected by C—H⋯O hydrogen bonds and weak C—H⋯π inter­actions help to consolidate the crystal packing.

## Related literature

For industrial applications of selenium, see: Stevenson (2011 ▶); Comasseto *et al.* (1997 ▶). For its biological function, see: Gladyshev *et al.* (1996 ▶); Epp *et al.* (1983 ▶); Wessjohann *et al.* (2007 ▶). 
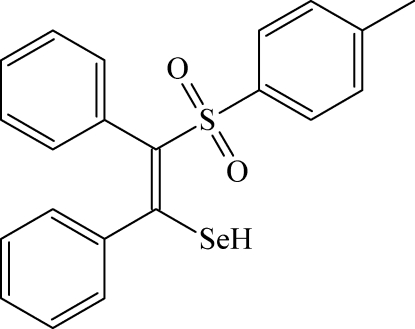


## Experimental

### 

#### Crystal data

C_21_H_18_O_2_SSe
*M* *_r_* = 413.37
Monoclinic, 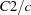

*a* = 21.601 (3) Å
*b* = 8.5238 (12) Å
*c* = 21.187 (3) Å
β = 106.175 (4)°
*V* = 3746.5 (9) Å^3^
*Z* = 8
Mo *K*α radiation
μ = 2.13 mm^−1^
*T* = 293 K
0.32 × 0.20 × 0.16 mm

#### Data collection

Bruker SMART APEX CCD area-detector diffractometer
20922 measured reflections
4449 independent reflections
3330 reflections with *I* > 2σ(*I*)
*R*_int_ = 0.029

#### Refinement

*R*[*F* ^2^ > 2σ(*F* ^2^)] = 0.040
*wR*(*F* ^2^) = 0.114
*S* = 1.01
4449 reflections
226 parameters
H-atom parameters constrained
Δρ_max_ = 0.65 e Å^−3^
Δρ_min_ = −0.28 e Å^−3^

### 

Data collection: *SMART* (Bruker, 2004 ▶); cell refinement: *SAINT* (Bruker, 2004 ▶); data reduction: *SAINT*; program(s) used to solve structure: *SHELXS97* (Sheldrick, 2008 ▶); program(s) used to refine structure: *SHELXL97* (Sheldrick, 2008 ▶); molecular graphics: *ORTEP-3* (Farrugia, 1997 ▶); software used to prepare material for publication: *PLATON* (Spek, 2009 ▶).

## Supplementary Material

Crystal structure: contains datablock(s) I, global. DOI: 10.1107/S1600536812001638/bq2332sup1.cif

Structure factors: contains datablock(s) I. DOI: 10.1107/S1600536812001638/bq2332Isup2.hkl

Supplementary material file. DOI: 10.1107/S1600536812001638/bq2332Isup3.cml

Additional supplementary materials:  crystallographic information; 3D view; checkCIF report

## Figures and Tables

**Table 1:** Hydrogen-bond geometry (Å, °) *Cg*1 is the centroid of the C9–C14 ring.

*D*—H⋯*A*	*D*—H	H⋯*A*	*D*⋯*A*	*D*—H⋯*A*
Se1—H1*A*⋯O2	0.82	2.57	3.141 (2)	128
C4—H4⋯O1^i^	0.93	2.75	3.557 (4)	145
C17—H17⋯O1^ii^	0.93	2.65	3.567 (3)	168
C5—H5⋯*Cg*1^iii^	0.93	2.92	3.615	132
C19—H19⋯*Cg*1^iv^	0.93	2.88	3.791	165

Symmetry codes: (i) 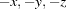; (ii) 
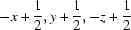
; (iii) 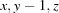; (iv) 
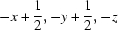
.

**Fig. 1.**
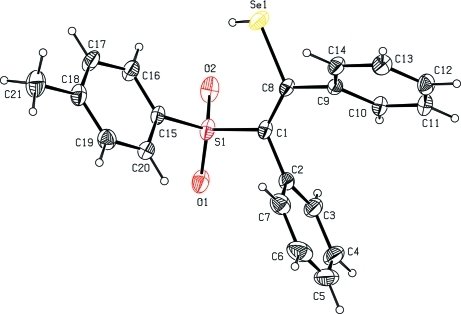
ORTEP diagram of the title molecule with the atom numbering scheme. Displacement ellipsoids are drawn at 30% probability level.

**Fig. 2.**
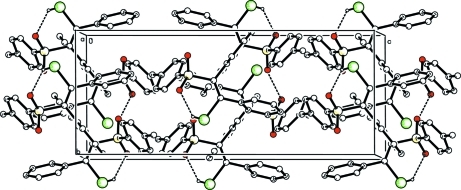
A unit cell packing of the crystal structure of the title compound viewed down a axis. The dashed lines indicate the hydrogen bonds.

## supplementary crystallographic information

### Comment

Selenium is chemically related to sulfur and oxygen and has a wide range of applications in the inorganic field, including uses in semiconductors, photovoltaic and photocell devices, and photographic toner applications. It is used industrially to produce deep red color in glasses and ceramics (Stevenson, 2011). Vinylic selenides are organoselenium compounds that play a role in organic synthesis, especially in the development of convenient stereoselective routes to functionalized alkenes (Comasseto *et al.*, 1997). Selenols have important roles in certain biological processes. Three enzymes namely iodothyronine deiodinase, glutathione peroxidase (Epp *et al.*, 1983) and thioredoxin reductase (Gladyshev *et al.*, 1996) found in mammals contain selenols at their active sites. The selenols in these proteins are part of the essential amino acid selenocysteine (Wessjohann *et al.*, 2007).

The title molecule is an organoselenium compound (Fig. 1) containing three phenyl rings with vinylic selenol group. The dihedral angle between the *cis* substituted phenyl rings is 64.3 (1)°. The dihedral angles between the toluene sufonyl and the phenyl rings substituted on C1 and C8 atoms are 21.1 (2)° and 72.0 (1)°, respectively. The molecules are not showing any classical hydrogen bonds, still the molecules accept a Se—H···O type intra (Fig.2) and two C—H···O type intermolecular interactions. In addition, two C—H···π interactions also help to consolidate the molecules in the unit cell crystal packing. The complete details of the molecular interactions are shown in Table 1.

### Experimental

The compound 2-phenylacetophenone (0.1*M*) was heated with Woollins' reagent (1 equiv.) in toluene to produce 2-phenylselenoacetophenone. Then 1 equiv. of this compound was stirred with *p*-toluenesulfonylchloride (1 equiv.) in the presence of triethylamine (2 equiv.) in dry THF resulted the title compound and was recrystallized using methanol.

### Refinement

H atoms were positioned geometrically and refined using a riding model with Se—H = 0.82Å for selenol H, C—H = 0.93 Å for aromatic H and 0.96 Å for methyl H atoms. The *U*_iso_ values were constrained to be 1.5Ueq of the carrier atom for the methyl and selenol H atoms and 1.2Ueq for the remaining H atoms.

### Crystal data

**Table:**

C_21_H_18_O_2_SSe	*F*(000) = 1680
*M**_r_* = 413.37	*D*_x_ = 1.466 Mg m^−^^3^
Monoclinic, *C*2/*c*	Mo *K*α radiation, λ = 0.71073 Å
Hall symbol: -C 2yc	Cell parameters from 4449 reflections
*a* = 21.601 (3) Å	θ = 2.0–28.4°
*b* = 8.5238 (12) Å	µ = 2.13 mm^−^^1^
*c* = 21.187 (3) Å	*T* = 293 K
β = 106.175 (4)°	Needle, colorless
*V* = 3746.5 (9) Å^3^	0.32 × 0.20 × 0.16 mm
*Z* = 8	

### Data collection

**Table:**

Bruker SMART APEX CCD area-detector diffractometer	3330 reflections with *I* > 2σ(*I*)
Radiation source: fine-focus sealed tube	*R*_int_ = 0.029
graphite	θ_max_ = 28.4°, θ_min_ = 2.0°
ω scans	*h* = −28→27
20922 measured reflections	*k* = −11→11
4449 independent reflections	*l* = −26→28

### Refinement

**Table:**

Refinement on *F*^2^	Primary atom site location: structure-invariant direct methods
Least-squares matrix: full	Secondary atom site location: difference Fourier map
*R*[*F*^2^ > 2σ(*F*^2^)] = 0.040	Hydrogen site location: inferred from neighbouring sites
*wR*(*F*^2^) = 0.114	H-atom parameters constrained
*S* = 1.01	*w* = 1/[σ^2^(*F*_o_^2^) + (0.0722*P*)^2^ + 0.6895*P*] where *P* = (*F*_o_^2^ + 2*F*_c_^2^)/3
4449 reflections	(Δ/σ)_max_ < 0.001
226 parameters	Δρ_max_ = 0.65 e Å^−^^3^
0 restraints	Δρ_min_ = −0.28 e Å^−^^3^

### Special details

**Table:**

Geometry. All e.s.d.'s (except the e.s.d. in the dihedral angle between two l.s. planes) are estimated using the full covariance matrix. The cell e.s.d.'s are taken into account individually in the estimation of e.s.d.'s in distances, angles and torsion angles; correlations between e.s.d.'s in cell parameters are only used when they are defined by crystal symmetry. An approximate (isotropic) treatment of cell e.s.d.'s is used for estimating e.s.d.'s involving l.s. planes.
Refinement. Refinement of *F*^2^ against ALL reflections. The weighted *R*-factor *wR* and goodness of fit *S* are based on *F*^2^, conventional *R*-factors *R* are based on *F*, with *F* set to zero for negative *F*^2^. The threshold expression of *F*^2^ > σ(*F*^2^) is used only for calculating *R*-factors(gt) *etc*. and is not relevant to the choice of reflections for refinement. *R*-factors based on *F*^2^ are statistically about twice as large as those based on *F*, and *R*- factors based on ALL data will be even larger.

### Fractional atomic coordinates and isotropic or equivalent isotropic displacement parameters (Å^2^)

**Table:**

	*x*	*y*	*z*	*U*_iso_*/*U*_eq_	
Se1	0.158447 (15)	0.71437 (4)	0.075900 (13)	0.06873 (13)	
H1A	0.1621	0.6889	0.1141	0.103*	
S1	0.12700 (3)	0.35507 (8)	0.12702 (3)	0.05289 (17)	
O1	0.10403 (10)	0.1978 (3)	0.12827 (9)	0.0747 (6)	
O2	0.09829 (8)	0.4776 (3)	0.15593 (8)	0.0696 (5)	
C1	0.11840 (10)	0.3982 (3)	0.04198 (10)	0.0450 (5)	
C2	0.09960 (12)	0.2622 (3)	−0.00303 (11)	0.0492 (5)	
C3	0.03661 (15)	0.2087 (3)	−0.02158 (14)	0.0642 (7)	
H3	0.0058	0.2576	−0.0053	0.077*	
C4	0.0192 (2)	0.0830 (4)	−0.06412 (15)	0.0872 (11)	
H4	−0.0233	0.0486	−0.0771	0.105*	
C5	0.0654 (3)	0.0093 (4)	−0.08708 (17)	0.1021 (13)	
H5	0.0541	−0.0761	−0.1153	0.123*	
C6	0.1279 (2)	0.0611 (4)	−0.06857 (18)	0.0959 (12)	
H6	0.1590	0.0110	−0.0842	0.115*	
C7	0.14454 (17)	0.1860 (3)	−0.02735 (15)	0.0698 (7)	
H7	0.1870	0.2207	−0.0153	0.084*	
C8	0.12986 (10)	0.5387 (3)	0.01975 (10)	0.0450 (5)	
C9	0.12153 (10)	0.5774 (3)	−0.05031 (10)	0.0438 (5)	
C10	0.06198 (11)	0.5576 (3)	−0.09579 (11)	0.0503 (5)	
H10	0.0274	0.5190	−0.0824	0.060*	
C11	0.05388 (12)	0.5951 (3)	−0.16109 (11)	0.0587 (6)	
H11	0.0139	0.5809	−0.1914	0.070*	
C12	0.10403 (14)	0.6527 (3)	−0.18150 (12)	0.0622 (6)	
H12	0.0981	0.6783	−0.2255	0.075*	
C13	0.16341 (14)	0.6730 (3)	−0.13693 (13)	0.0619 (6)	
H13	0.1976	0.7119	−0.1509	0.074*	
C14	0.17247 (11)	0.6355 (3)	−0.07140 (12)	0.0533 (6)	
H14	0.2127	0.6493	−0.0415	0.064*	
C15	0.21090 (10)	0.3538 (3)	0.16456 (10)	0.0463 (5)	
C16	0.23804 (12)	0.4644 (3)	0.21091 (12)	0.0612 (6)	
H16	0.2128	0.5427	0.2217	0.073*	
C17	0.30360 (13)	0.4579 (4)	0.24136 (13)	0.0683 (7)	
H17	0.3221	0.5317	0.2734	0.082*	
C18	0.34198 (12)	0.3450 (4)	0.22527 (12)	0.0597 (6)	
C19	0.31359 (14)	0.2362 (3)	0.17798 (15)	0.0641 (7)	
H19	0.3391	0.1599	0.1662	0.077*	
C20	0.24825 (13)	0.2378 (3)	0.14778 (13)	0.0561 (6)	
H20	0.2296	0.1622	0.1166	0.067*	
C21	0.41369 (15)	0.3404 (5)	0.25779 (18)	0.0914 (10)	
H21A	0.4324	0.2544	0.2404	0.137*	
H21B	0.4214	0.3270	0.3043	0.137*	
H21C	0.4328	0.4370	0.2494	0.137*	

### Atomic displacement parameters (Å^2^)

**Table:**

	*U*^11^	*U*^22^	*U*^33^	*U*^12^	*U*^13^	*U*^23^
Se1	0.0826 (2)	0.0716 (2)	0.04728 (17)	−0.02695 (14)	0.01026 (14)	−0.01444 (11)
S1	0.0415 (3)	0.0792 (4)	0.0358 (3)	−0.0115 (3)	0.0071 (2)	0.0064 (3)
O1	0.0680 (12)	0.0960 (16)	0.0515 (10)	−0.0343 (10)	0.0026 (9)	0.0196 (9)
O2	0.0500 (9)	0.1144 (16)	0.0488 (9)	0.0079 (10)	0.0210 (8)	0.0008 (10)
C1	0.0353 (10)	0.0628 (13)	0.0346 (10)	−0.0058 (9)	0.0061 (8)	0.0020 (9)
C2	0.0570 (13)	0.0507 (12)	0.0383 (11)	−0.0034 (10)	0.0107 (10)	0.0070 (9)
C3	0.0683 (16)	0.0668 (17)	0.0528 (15)	−0.0167 (13)	0.0087 (13)	−0.0056 (12)
C4	0.117 (3)	0.074 (2)	0.0586 (17)	−0.041 (2)	0.0050 (18)	−0.0043 (15)
C5	0.194 (5)	0.0526 (17)	0.0610 (18)	−0.025 (2)	0.037 (2)	−0.0074 (14)
C6	0.159 (4)	0.061 (2)	0.083 (2)	0.014 (2)	0.060 (3)	0.0005 (17)
C7	0.0821 (19)	0.0677 (18)	0.0652 (17)	0.0075 (14)	0.0297 (15)	0.0082 (13)
C8	0.0368 (10)	0.0551 (13)	0.0391 (10)	−0.0070 (9)	0.0040 (8)	−0.0043 (9)
C9	0.0458 (11)	0.0452 (11)	0.0380 (10)	−0.0038 (9)	0.0079 (9)	−0.0012 (9)
C10	0.0426 (11)	0.0632 (14)	0.0420 (11)	−0.0011 (10)	0.0067 (9)	0.0020 (10)
C11	0.0521 (13)	0.0794 (17)	0.0389 (12)	0.0064 (12)	0.0032 (10)	−0.0006 (11)
C12	0.0751 (17)	0.0743 (17)	0.0377 (12)	0.0038 (14)	0.0167 (12)	0.0014 (11)
C13	0.0653 (15)	0.0708 (16)	0.0548 (14)	−0.0113 (13)	0.0253 (12)	−0.0009 (12)
C14	0.0496 (12)	0.0612 (15)	0.0460 (12)	−0.0124 (11)	0.0082 (10)	−0.0036 (10)
C15	0.0418 (11)	0.0613 (13)	0.0324 (10)	−0.0050 (10)	0.0044 (8)	0.0025 (9)
C16	0.0545 (14)	0.0766 (17)	0.0467 (13)	0.0072 (12)	0.0045 (11)	−0.0137 (12)
C17	0.0558 (14)	0.0840 (19)	0.0525 (14)	−0.0063 (14)	−0.0059 (11)	−0.0184 (13)
C18	0.0450 (12)	0.0784 (17)	0.0503 (13)	−0.0020 (12)	0.0044 (10)	0.0105 (13)
C19	0.0594 (15)	0.0687 (16)	0.0628 (17)	0.0113 (13)	0.0143 (13)	0.0015 (13)
C20	0.0597 (15)	0.0565 (14)	0.0471 (13)	−0.0021 (11)	0.0065 (11)	−0.0025 (10)
C21	0.0499 (15)	0.124 (3)	0.089 (2)	0.0031 (17)	0.0002 (15)	0.009 (2)

### Geometric parameters (Å, °)

**Table:**

Se1—C8	1.905 (2)	C10—H10	0.9300
Se1—H1A	0.8200	C11—C12	1.365 (4)
S1—O1	1.432 (2)	C11—H11	0.9300
S1—O2	1.436 (2)	C12—C13	1.375 (4)
S1—C15	1.765 (2)	C12—H12	0.9300
S1—C1	1.797 (2)	C13—C14	1.384 (4)
C1—C8	1.335 (3)	C13—H13	0.9300
C1—C2	1.484 (3)	C14—H14	0.9300
C2—C7	1.382 (4)	C15—C16	1.370 (3)
C2—C3	1.384 (4)	C15—C20	1.384 (4)
C3—C4	1.383 (4)	C16—C17	1.384 (4)
C3—H3	0.9300	C16—H16	0.9300
C4—C5	1.378 (6)	C17—C18	1.374 (4)
C4—H4	0.9300	C17—H17	0.9300
C5—C6	1.370 (6)	C18—C19	1.378 (4)
C5—H5	0.9300	C18—C21	1.510 (4)
C6—C7	1.360 (5)	C19—C20	1.378 (4)
C6—H6	0.9300	C19—H19	0.9300
C7—H7	0.9300	C20—H20	0.9300
C8—C9	1.482 (3)	C21—H21A	0.9600
C9—C10	1.386 (3)	C21—H21B	0.9600
C9—C14	1.389 (3)	C21—H21C	0.9600
C10—C11	1.383 (3)		
			
C8—Se1—H1A	109.5	C12—C11—C10	120.6 (2)
O1—S1—O2	118.66 (13)	C12—C11—H11	119.7
O1—S1—C15	107.67 (12)	C10—C11—H11	119.7
O2—S1—C15	108.88 (11)	C11—C12—C13	119.9 (2)
O1—S1—C1	105.69 (11)	C11—C12—H12	120.1
O2—S1—C1	110.04 (11)	C13—C12—H12	120.1
C15—S1—C1	105.04 (10)	C12—C13—C14	120.3 (2)
C8—C1—C2	121.23 (19)	C12—C13—H13	119.9
C8—C1—S1	124.04 (17)	C14—C13—H13	119.9
C2—C1—S1	114.69 (16)	C13—C14—C9	120.1 (2)
C7—C2—C3	118.4 (3)	C13—C14—H14	119.9
C7—C2—C1	120.8 (2)	C9—C14—H14	119.9
C3—C2—C1	120.8 (2)	C16—C15—C20	120.8 (2)
C4—C3—C2	120.5 (3)	C16—C15—S1	119.97 (19)
C4—C3—H3	119.7	C20—C15—S1	119.19 (18)
C2—C3—H3	119.7	C15—C16—C17	119.0 (2)
C5—C4—C3	119.4 (3)	C15—C16—H16	120.5
C5—C4—H4	120.3	C17—C16—H16	120.5
C3—C4—H4	120.3	C18—C17—C16	121.5 (2)
C6—C5—C4	120.3 (3)	C18—C17—H17	119.3
C6—C5—H5	119.8	C16—C17—H17	119.3
C4—C5—H5	119.8	C17—C18—C19	118.4 (2)
C7—C6—C5	119.9 (4)	C17—C18—C21	121.2 (3)
C7—C6—H6	120.0	C19—C18—C21	120.4 (3)
C5—C6—H6	120.0	C18—C19—C20	121.4 (3)
C6—C7—C2	121.4 (3)	C18—C19—H19	119.3
C6—C7—H7	119.3	C20—C19—H19	119.3
C2—C7—H7	119.3	C19—C20—C15	118.9 (2)
C1—C8—C9	124.8 (2)	C19—C20—H20	120.6
C1—C8—Se1	122.99 (16)	C15—C20—H20	120.6
C9—C8—Se1	112.23 (16)	C18—C21—H21A	109.5
C10—C9—C14	119.0 (2)	C18—C21—H21B	109.5
C10—C9—C8	119.96 (19)	H21A—C21—H21B	109.5
C14—C9—C8	121.05 (19)	C18—C21—H21C	109.5
C11—C10—C9	120.1 (2)	H21A—C21—H21C	109.5
C11—C10—H10	119.9	H21B—C21—H21C	109.5
C9—C10—H10	119.9		
			
O1—S1—C1—C8	174.4 (2)	Se1—C8—C9—C14	57.8 (3)
O2—S1—C1—C8	45.2 (2)	C14—C9—C10—C11	0.1 (4)
C15—S1—C1—C8	−71.9 (2)	C8—C9—C10—C11	179.3 (2)
O1—S1—C1—C2	−7.9 (2)	C9—C10—C11—C12	−0.4 (4)
O2—S1—C1—C2	−137.13 (17)	C10—C11—C12—C13	0.4 (4)
C15—S1—C1—C2	105.83 (18)	C11—C12—C13—C14	−0.2 (4)
C8—C1—C2—C7	74.7 (3)	C12—C13—C14—C9	−0.1 (4)
S1—C1—C2—C7	−103.1 (2)	C10—C9—C14—C13	0.1 (4)
C8—C1—C2—C3	−105.4 (3)	C8—C9—C14—C13	−179.1 (2)
S1—C1—C2—C3	76.9 (3)	O1—S1—C15—C16	−132.8 (2)
C7—C2—C3—C4	−0.8 (4)	O2—S1—C15—C16	−2.9 (2)
C1—C2—C3—C4	179.3 (2)	C1—S1—C15—C16	114.9 (2)
C2—C3—C4—C5	1.2 (5)	O1—S1—C15—C20	45.6 (2)
C3—C4—C5—C6	−0.8 (5)	O2—S1—C15—C20	175.43 (19)
C4—C5—C6—C7	0.0 (5)	C1—S1—C15—C20	−66.7 (2)
C5—C6—C7—C2	0.4 (5)	C20—C15—C16—C17	−0.6 (4)
C3—C2—C7—C6	0.0 (4)	S1—C15—C16—C17	177.8 (2)
C1—C2—C7—C6	179.9 (3)	C15—C16—C17—C18	1.2 (4)
C2—C1—C8—C9	4.0 (3)	C16—C17—C18—C19	−0.5 (4)
S1—C1—C8—C9	−178.47 (17)	C16—C17—C18—C21	178.8 (3)
C2—C1—C8—Se1	−176.64 (16)	C17—C18—C19—C20	−0.9 (4)
S1—C1—C8—Se1	0.9 (3)	C21—C18—C19—C20	179.8 (3)
C1—C8—C9—C10	58.1 (3)	C18—C19—C20—C15	1.5 (4)
Se1—C8—C9—C10	−121.4 (2)	C16—C15—C20—C19	−0.7 (4)
C1—C8—C9—C14	−122.7 (3)	S1—C15—C20—C19	−179.1 (2)

### Hydrogen-bond geometry (Å, °)

**Table:**

Cg1 is the centroid of the C9–C14 ring.

**Table:**

*D*—H···*A*	*D*—H	H···*A*	*D*···*A*	*D*—H···*A*
Se1—H1A···O2	0.82	2.57	3.141 (2)	128
C4—H4···O1^i^	0.93	2.75	3.557 (4)	145
C17—H17···O1^ii^	0.93	2.65	3.567 (3)	168
C5—H5···Cg1^iii^	0.93	2.92	3.615	132
C19—H19···Cg1^iv^	0.93	2.88	3.791	165

Symmetry codes: (i) −*x*, −*y*, −*z*; (ii) −*x*+1/2, *y*+1/2, −*z*+1/2; (iii) *x*, *y*−1, *z*; (iv) −*x*+1/2, −*y*+1/2, −*z*.
